# Improving health Professional’s knowledge of hepatitis B using cartoon based learning tools: a retrospective analysis of pre and post tests

**DOI:** 10.1186/s12909-014-0244-7

**Published:** 2014-11-21

**Authors:** Moira G Sim, Ashleigh C McEvoy, Toni D Wain, Eric L Khong

**Affiliations:** Systems and Intervention Research Centre for Health, School of Medical Sciences, Edith Cowan University, 270 Joondalup Drive, Joondalup, 6027 WA Australia

**Keywords:** Medical education, Postgraduate training, Illustration, Visual learning, Cartoons

## Abstract

**Background:**

Hepatitis B serology is complex and a lack of knowledge in interpretation contributes to the inadequate levels of screening and referral for highly effective hepatitis antiviral treatments. This knowledge gap needs to be addressed so that current and future healthcare professionals are more confident in the detection and assessment of hepatitis B to improve the uptake of treatment and reduce long-term complications from the disease. Cartoons have been used effectively as a teaching tool in other settings and were considered as a potentially useful teaching aid in explaining hepatitis B serology. This study examines the impact of cartoons in improving healthcare professionals’ knowledge.

**Methods:**

A cartoon based learning tool designed to simplify the complexities of hepatitis B serology was developed as part of an online learning program for medical practitioners, nurses and students in these professions. A retrospective analysis was carried out of pre and post online test results.

**Results:**

An average improvement of 96% of correct answers to case study questions in hepatitis B serology was found across all ten questions following the use of an online cartoon based learning tool.

**Conclusion:**

The data indicates a significant improvement of participants’ knowledge of hepatitis B serology from pre-test to post-test immediately following an online cartoon based learning tool. However, further research is required to measure its long term impact.

**Electronic supplementary material:**

The online version of this article (doi:10.1186/s12909-014-0244-7) contains supplementary material, which is available to authorized users.

## Background

Chronic hepatitis B is a global public health concern given its association with increased burden of disease and premature death from liver cancer, cirrhosis and liver failure. Chronic hepatitis B virus infections are ranked as the tenth leading cause of deaths in the world [1]. Despite the availability of a highly effective vaccine and improvements in antiviral treatments, hepatitis B disease detection is suboptimal. In 2011 in Australia 209,000 people were estimated to have chronic hepatitis B [2] of which one third were undiagnosed and the incidence expected to triple by 2017 [3,4]. In the United States it is estimated that between 20 to 30% of those infected with the virus are aware of their infection [5].

Given that late diagnosis is associated with higher mortality and morbidity, appropriate screening and diagnostic testing in the primary healthcare setting is a key strategy in reducing the impact of hepatitis B infection [6].

In the last decade, there has been a considerable improvement in treatment regimen and outcomes for those with hepatitis B in clinical practice. The challenge of such evolution however, is to ensure that those in contact with patients remain current and confident in their knowledge [7]. Regrettably, routine screening remains low [8] and several studies have shown that general practitioners acknowledge the need to expand their knowledge and skills associated with hepatitis B [4,9,10].

Hepatitis B infections are detected via serological testing and a clear understanding of the different antigens and antibodies is crucial to accurate assessment of the disease stage and severity which precedes appropriate management. There are five serological markers which need to be interpreted together with the clinical picture and other laboratory tests: surface antigen (HBsAg), surface antibody (anti-HBs), core antibody (anti-HBc), e antigen (HBeAg) and e antibody (anti-HBe) [11,12]. However confusion associated with the interpretation of hepatitis B serology has been reported and general practitioners frequently refer to specialists for assistance with interpretation of results [7]. In one study 29.5% of general practitioners referred patients to a specialist following a positive hepatitis B surface antibody (anti-HBs) result, despite this indicating a resolved infection or successful vaccination [7]. This is understandable given that in other diseases, such as hepatitis C, the presence of the antibody does indeed usually signify the presence of infection. The complexity in hepatitis B serology with multiple antigens and antibodies creates the conditions for misinterpretation.

Effective treatments for hepatitis B are relatively new and were not available when the majority of current practising physicians underwent their medical training. Until the past decade, chronic HbsAg carriers were often referred to as “healthy carriers” with minimal liver disease for which active therapy was not widely recommended. In an editorial in the New England Journal of Medicine in 2006 Hoofnagle noted that hepatitis B had become a treatable disease when a landmark paper, published in 2004, demonstrated improvement in long-term outcomes. However he cautioned the need to be selective on who should have treatment [13]. It is therefore not surprising that general practitioners have been described as being passive with regards to screening and referring hepatitis B cases [9].

Although the number of hepatitis B virus infected patients can represent a small proportion of general practitioners’ patients, chronic hepatitis B is responsible for the leading cause of hepatocellular carcinoma (HCC) worldwide. To encourage healthcare practitioners to further develop their knowledge about hepatitis B infections and to optimise its screening and diagnosis in primary healthcare, novel educational methods are required. Since continuing medical education increases general practitioners clinical proficiency as well as improving the quality of healthcare services [14], education should extend to current and future primary care practitioners.

For many years, comics have been regarded as a valuable teaching aid [15] grounded on the dual coding theory which suggests that the combination of imagery and language improves cognitive operation with enhanced recall [16]. The scarcity of words often depicted in cartoons, forces the reader to interpret and complete the missing words, thereby producing an aesthetic response and increasing the readers’ involvement [17]. Furthermore, the space between the panels of each picture (referred to as the gutter) provides an opportunity for the reader to integrate the pictures, and convert several images into one idea. Whilst there is nothing between the pictures, experience tells us that there are [17]. McCloud suggests that it is the readers’ involvement and interpretation that allows for the transitions between one panel and another to be meaningful; the transition might not make immediate sense but a relationship can be developed in the readers’ minds. This lack of clarity fosters a greater participation by the reader, thereby increasing their cognitive state [17]. Notwithstanding the positive cognitive effects of cartoons, several authors have reported increased motivation, interest and dialogue when cartoons were integrated into the curriculum [15,18].

This medium, referred to as cartoons [17,19,20], graphic stories [15,17,20], or sequential art [17,20], has been used extensively in educating young people in areas such as literacy [21], science related topics [22], reading comprehension [23], ethics [24] and successfully used in patient care as well as promoting public awareness of diseases such as hepatitis C [25], diabetes [26] and human immunodeficiency virus [27] and increasing awareness on issues such as burn safety [28]. Despite the increasing use of cartoons in many areas of education empirical research is lacking with some studies showing subjective results [22]. In 2007, Weitkamp and Burnet found an increased understanding of science principles in secondary students due to the use of comics, however as the measurement of learning was conducted in a group setting, the applicability to individual students is unknown [22]. Recently Hosler and Boomer [29] considered the efficacy of a comic book to engage non-scientific major students in learning and appreciating science. A comic book was used as standalone text in one course or in conjunction with additional texts in other courses. Assessments at the start and at the end of the courses concluded that whilst there was a significant improvement in knowledge, the candidates would have been expected to have some form of increased knowledge regardless of the instruction material; the impact of the comic book on learning could not be isolated from the rest of the course content. It was recommended that future research should include a more controlled experimental design [29]. Moreover, these studies assessed undergraduate students in a science-based fraternity and did not include graduate students or professionals.

Other studies have shown that combining text with images increases not only reading performance but also retention when compared with non-illustrated texts [30–32]. In considering the specific utility of comics and cartoons as a method of learning, several studies have documented their value [23,33,34]. However, only one study recovered through the literature search has specifically evaluated the effectiveness of the cartoon concept in medical education. In 1974 Kauffman and Dwyer [34] investigated the efficacy of different forms of visual illustrations on medical information retention in three different populations: (1) instructional media undergraduate students, (2) state employees without an undergraduate or nursing degree and (3) state employees with an undergraduate or nursing degree. Interpretation of the statistical evidence obtained from this study showed that in both immediate and four-week retention recall cartoon presentation was effective. Furthermore, the majority of subjects not only learned more from instruction complemented by cartoons, but also preferred this medium.

Green and Myers [15] suggest that combining pictures with text is a new cultural trend that can be integrated into medical training as they can convey messages in ways that conventional texts cannot; the visual images in the cartoons, combined with the text activate different processing systems in the brain which have been shown to improve understanding [35] and increase recall of medical information [36]. Furthermore, embedding text into an image (rather than having the text and image separated) fosters a greater understanding of a topic [37]. With this in mind and since no research, to our knowledge, has specifically evaluated the efficacy of cartoons in hepatitis B serology education, an opportunity to increase healthcare professionals’ knowledge on hepatitis B serology by way of cartoons was considered a novel and promising medium.

## Methods

### Design

This was a quasi-experimental study with pre-test and post-test design. The participants were recruited between February 2010 and January 2013.

### Setting

In a project funded by the Sexual Health and Blood-Borne Virus Program, Department of Health, Western Australia, to enhance healthcare professionals’ knowledge in the assessment and management of patients with hepatitis B and to improve access to treatment for these patients, a comprehensive hepatitis B online training program was developed using a combination of text, illustrations, diagrams, tables and case studies.

### Cartoons

A cartoon based learning tool (CBL) was subsequently developed by two of the authors (MGS and ELK). Being primary care physicians/general practitioners themselves, they recognize that hepatitis serology can be confusing because of the number of serological markers involved. The main learning objective is to improve understanding of hepatitis B serology. These markers provide information about whether the virus is still in the body, if the body is immune and if the virus is active. Key learning objectives for the CBL were developed by the authors (MGS and ELK) and a hepatologist informed by discussions with fellow general practitioners, medical students and other hepatology specialists.

Considering cartoons to be a novel and potential medium to convey these difficult concepts a series of cartoons were developed as an aide memoire which encompass different characters that represent the various components of hepatitis B serology. The appearance of each cartoon character was conceptually simple and modelled on the qualities of the various antigens and antibodies. Combining the image with the name and serological marker simplifies the concept. The characters are divided into two groups and using the classic story line of good versus evil, alien invaders attempt to subdue vulnerable earthlings.

Under the guise of evil characters, the nonhuman beings are personified: alien forces represent the antigens of the hepatitis B virus. A spaceship represents the HBsAg antigen, the commander of the alien forces represents HBcAg and enemy soldiers represent HBeAg where the differing letter stands for ‘spaceship’, ‘commander’ and ‘enemy’ respectively, providing a memory prompt within the name. Similarly, the earth’s forces which must come to the rescue represent the human body’s primary form of defence, the antibodies. Again the differing letter of the antigen stands for the earth’s ‘commander’, the ‘special air-force’ and ‘earth fighters’, anti-HBc, anti-HBs and anti-HBe, respectively.

**Figure 1:**
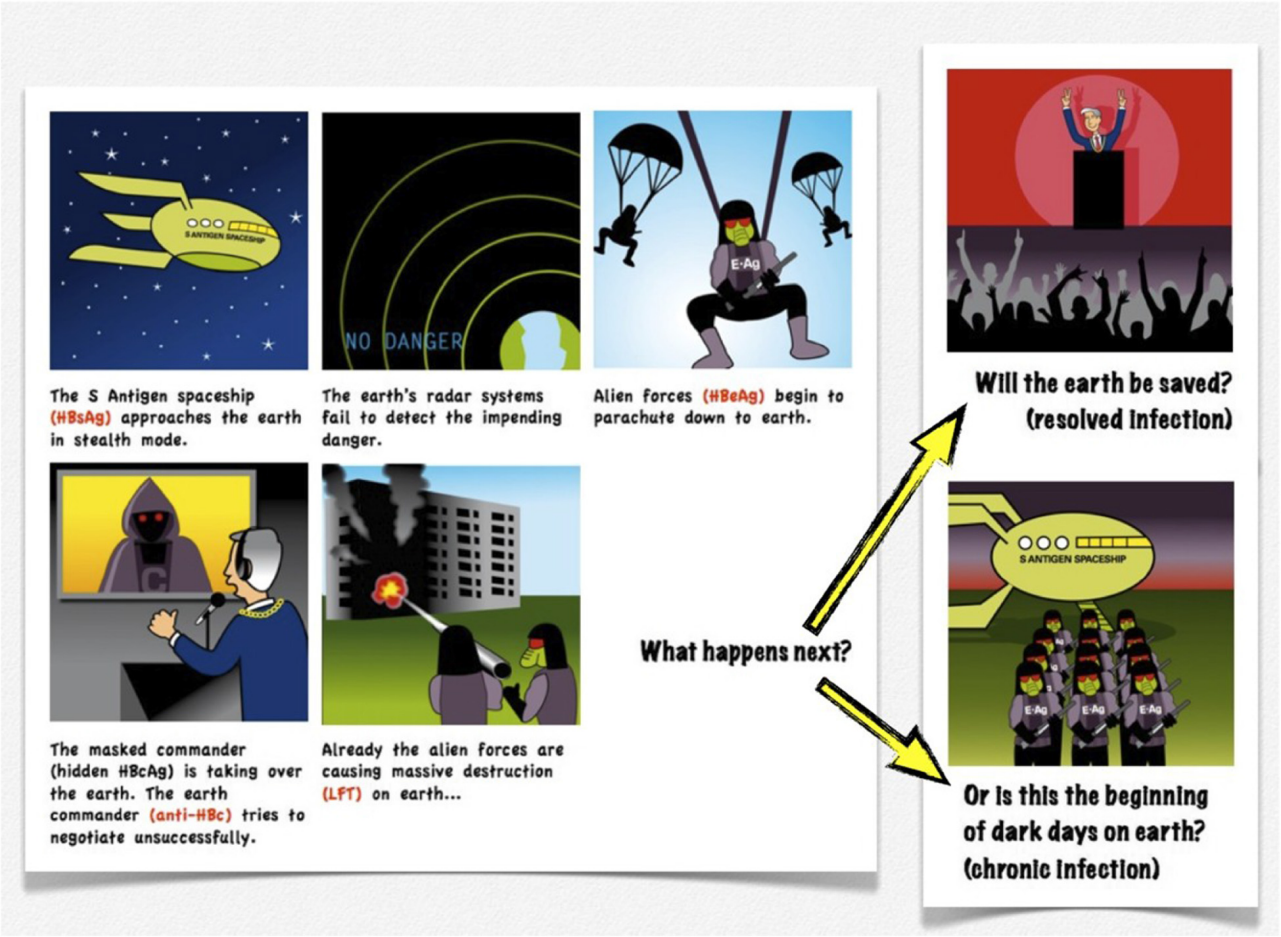
**A page from** ***Cartoons to the rescue: Understanding hepatitis B serology*** **in which the S Antigen spaceship approaches earth depositing enemy forces (HBeAg) to depict acute hepatitis infection.**

Embedding the characters into a series of comic strips provided a platform to explain the different stages of the disease. Each story demonstrates a possible progression should a specific pathway be followed such as a simple concept of how immunisation can prevent hepatitis, or more complex scenarios where an infection is resolved, deteriorates into chronic infection or co-exists with the host. The cartoon series can be accessed at http://hepatitis.ecu.edu.au/hepb/cartoons/index.php (Figure 1).

### Participants

The free online hepatitis B learning program provided the opportunity to recruit participants. 699 healthcare professionals who had completed the online hepatitis B training program undertook an online pre-test consisting of questions relating to the serology of hepatitis B before viewing the CBL tool. After completing the CBL tool 321 participants, from the original 699 cohort, voluntarily completed the online post-test. The pre-and post-tests were anonymous but participants were asked to provide their year of birth and the first two letters of their mother’s maiden name to assist in pre and post-test matching. Since the questionnaires were anonymous, written informed consent was not obtained and response to questionnaires was implied as verbal consent. Results from 201 post-tests were excluded due to: multiple attempts by the same participant, unmatched pre and post-test and incomplete responses. This resulted in a total of 120 participants being included in the study: 82 described themselves as medical students, 19 as medical practitioners, 3 as nurses, 1 as a nursing student, 3 as allied health and 12 as other. Based on the small quantity of participants in the nursing and allied health fields, these groups were amalgamated and described as ‘other’ for reporting purposes.

### Pre and post test

Participants’ knowledge was evaluated using a multiple-choice pre and post-test consisting of the same ten questions relating to the serology of hepatitis B including viral specific antigens and antibodies, determining the most appropriate tests required, stages of infection and interpretation of results using realistic case studies. (Refer to Additional file 1 for the full set of questions). Two general practitioners (MGS and ELK) each with over 20 years of clinical experience established the content validity of the test and these were checked by a hepatologist in Royal Perth Hospital, Professor Wendy Cheng. Scores for each pre and post-test ranged from 0 to 10 points: 1 point for each correct answer. Following the post-test, participants were asked to provide comments and suggestions for improvement relating to the cartoons.

### Analysis

The difference between a participants’ scores (correct responses) for the pre and post-test determined the level of knowledge gained (if any) following exposure to the CBL tool.

Data was analysed using R 3.0.2 and Microsoft Excel:

A linear mixed model analysis with AIC model fitting with R using R packages nlme and AICcmodavg; summary statistics; descriptive statistics and Student’s paired *t*-Test in Excel to compare the scores before and after the cartoon based learning tool intervention overall and within groups. The level of significance was set at 0.05 for all tests.

### Ethics

Ethics approval was granted by Edith Cowan University Human Ethics Committee (reference number 8928).

## Results

Due to group substructure and the repeated measures nature of the pre-post design, a linear mixed model (LME) was constructed to test overall statistical significance of the differences between pre and post CBL tool scores. With “Test-Score” as the response variable, individual ID as a random effect as each individual was sampled twice. This repeated sampling was represented as a “Pre-Post” factor effect with two types “Before” or “After” representing the two scores for each ID. An additional “Group” fixed affect was also included in the model as each individual was a member of one of three groups (Medical Practitioner, Medical Student or Other).

Various LME model structures were constructed with R package nlme both with and without fixed effect factor interactions. These models were tested with R package AICcmodavg with the model with smallest AIC score selected.

The best fitting model involved an interaction between the two fixed effects. The final model concept, in nlme formula, was:

$$ \mathrm{Test}\_\mathrm{Score} \sim \mathrm{Group}*\mathrm{PrePost},\ \mathrm{Random} = \sim 1/\mathrm{ID} $$

A Q-Q plot of the model showed an approximate straight line so we could conclude that the data from the model represents that from a normal population thus the LME was viable.

The ANOVA table of the LME (Table 1) shows a significant relationship between “Pre-Post” and test scores as well as a significant relationship between the interaction of group membership and “Pre-Post” and test score. However group membership on its own had no significant effect on test score changes.

**Table 1:** **ANOVA table for the linear mixed model**

	**numDF**	**denDF**	**F-value**	**p-value**
**Intercept**	1	117	2620.7331	<0.0001
**Group**	2	117	2.0231	0.1369
**Pre-post**	1	117	204.9999	<0.0001
**Group: pre-post**	2	117	5.4655	0.0054

Therefore test scores pre and post CBL tool are significantly different overall as well as within groups.

**Figure 2:**
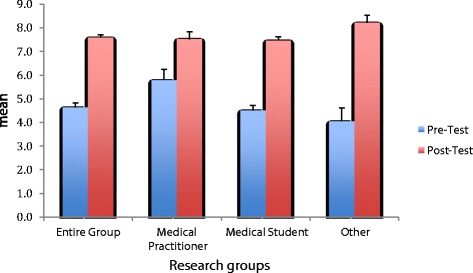
**Pre and post-test results.** Values represent the mean ± SEM.

Further investigations of the nature of the test score changes overall and within groups and for questions were conducted with a series of paired t-tests and descriptive statistics. Overall, the data indicate significant improvement of participants’ scores in the post-test immediately following the CBL tool. As shown in Figure 2, the entire group of participants showed significantly higher knowledge (mean = 7.6, standard deviation = 1.4) after completion of the CBL tool, compared to former knowledge (mean = 4.6, standard deviation = 2.1), *t*(120) = 13.809, *P* = 0.000. The mean pre-post test differences in the “other” group (mean =4.1, standard deviation =3.0 *t*(19) = 6.107, *P* =0.000) however showed more gains, than medical students (mean =3.0, standard deviation = 2.2 t(82) = 12.309, *P* = 0.000) and medical practitioners (mean = 1.7, standard deviation = 1.8 *t*(19) =4.304, *P* = 0.000).

Questions 1, 4, 5, 6 and 8 were correctly answered by the majority of participants in the pre-test (84%, 59%, 80%, 59% and 59% respectively). This is not surprising as these relate to the detection or exclusion of current infection, resolution of infection and effective immunisation, all of which are common roles in primary care, and relevant to healthcare professionals and students as they usually have to undergo testing for hepatitis B and/or immunisation. The remaining questions relate to the detection of chronic infection and identification of the phases of chronic infection which are relatively recent developments in knowledge. Only 20%, 22%, 37%, 25% and 19% answered the pre-test questions correctly in each of these questions. Until relatively recently effective treatments were not widely accessible and therefore the identification of phases of chronic hepatitis B were of little relevance to clinical management outside of a hepatology specialist context. In recent years that the concept of the “healthy” or inactive carrier (chronic hepatitis B infection) has been discouraged as it promotes passivity in management and does not recognise that asymptomatic progressive liver disease can occur [38].

**Figure 3:**
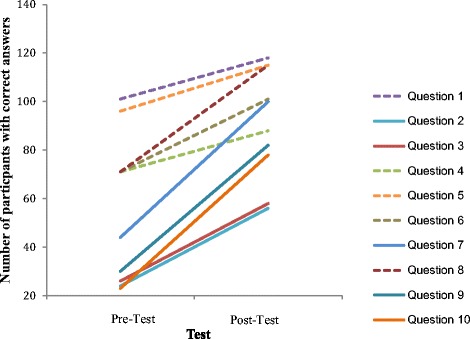
**A trajectory plot showing the changes in knowledge gain across individual questions by category.** Dashed lines relate to detection/exclusion of current infection. Solid lines relate to detection and identification of phases of chronic infection.

As expected the greatest knowledge gain was in the questions pertaining to phases of chronic hepatitis: 239% (question 10) and 173% (question 9). Conversely the least knowledge gained was in two questions most frequently answered correctly in the pre-test: 17% (question 1, correct in 84% of the pre-test scores) and 20% (question 5, correct in 80% of the pre-test scores) where there was little room for improvement. The average percentage increase was 96% across all ten questions. Figure 3 depicts the total number of questions answered correctly in each pre- and post-test visually emphasising the knowledge gained in each question.

A small number of participants scored lower in the post-test than in the pre-test: medical student (n = 2), medical practitioner (n =4), “other” (n = 1). Of all participants 4.1% (n =5) scored 1 point lower, 1.6% (n =2) scored 2 points lower and 0.8% (n =1) scored 4 points lower in the post-test than the pre-test.

In regards to the written response, participants were asked to provide comments and suggestions for improvement about the cartoons. 55.8% of the study participants (n =67) completed this section, all (except one) with positive comments but none with negative comments. This qualitative data was insufficient for further analysis.

## Discussion

This study assessed the effectiveness of a cartoon based learning tool to increase healthcare practitioners’ knowledge on hepatitis B serology. The data indicates that the use of a cartoon based learning tool was successful and aligns with the proposition that cartoons are a valuable teaching aid in medicine [15]. The authors acknowledge that the online training module includes a substantial amount of information on hepatitis B serology and therefore, participants would be expected to gain some knowledge before completing the pre-test and before being exposed to the cartoons. The effect of the other information provided would have minimised the effect of the cartoons and it is possible that without the additional information provided the effect of the cartoons would have been even greater. In addition, since the post-test was completed by the majority of participants (96.7%, n = 116) on the same day as the pre-test and immediately after exposure to the cartoon tool, we suggest that the additional knowledge was gained predominantly as a result of the exposure to the cartoon tool, ensuring a more specifically controlled design than reported by others [29]. We propose that the cartoons helped participants to retain the information particularly when they needed to think through the implications of serology presented in the case studies. Furthermore, participants who commented on the use of cartoons as a learning tool stated that the experience was useful (94%) (n =63) and reported an increased understanding of hepatitis B serology (22%) (n =15). Regrettably, because this was an optional, anonymous question, the researchers were unable to further analyse this group. The success of many learning intervention programs is largely affected by participants’ attitudes to the opportunities that are provided [39] and as such future research would benefit from specifically asking participants if and if so how they found the learning tool to be beneficial.

It is unclear as to why a small group scored lower in the post-test compared to the pre-test. This may reflect inattention or boredom having been confronted with the same questions as presented in the pre-test. Educators are aware that varying outcomes to learning methodologies are common, however future studies will benefit from including Likert-type attitude scales (e.g. totally focussed, partly focused, completely unfocused) to indicate the subjects’ focus whilst completing the tests [40].

There are several noteworthy limitations to this study. Without a control group, it is not clear if the significant increase in knowledge was due solely to the cartoon methodology or if it was simply due to the educational activity. It is recommended therefore that further studies be conducted with the inclusion of a control group who are not exposed to the CBL tool but only the standard online training program. This study does not assess any long-term knowledge retention and we recommend that future studies allow for a fixed time frame between last viewing of the cartoon and the post-test. It is also not clear how the detail of the cartoon design impacted on learning. Future studies may need to consider the use of black and white in comparison to colourful cartoons as well as the use of speech bubbles rather than explanatory text since Mayer [37] has shown that inserting text into an image results in a better understanding of a topic.

## Conclusion

An on-line cartoon based learning tool designed to simplify the complexities of hepatitis B serology was found to be effective in increasing healthcare professionals’ knowledge in hepatitis B serology. These findings not only add to the current body of literature which suggests that cartoons are an effective teaching aid, but also provide a novel educational method that will offer increased hepatitis B serological knowledge amongst healthcare professionals which will ultimately optimise early diagnosis and treatment of patients with hepatitis B. Whilst the results show an improvement in scores immediately after the cartoon based learning tool, it is important to note that long-term knowledge retention has not been considered and hence further analysis to assess such impacts is warranted.

## Additional file

Additional file 1:
**Questions used in the pre and post tests.**

## Abbreviations

HCC: Hepatocellular carcinoma
CBL: Cartoon based learning
LME: Linear mixed model
