# Supplementary material for: Improving health Professional’s knowledge of hepatitis B using cartoon based learning tools: a retrospective analysis of pre and post tests
Source: BMC Med Educ. 2014 Nov 21;14:244. doi: 10.1186/s12909-014-0244-7 (PMC4243383; doi:10.1186/s12909-014-0244-7)
Supplement: Additional file 1: — Questions used in the pre and post tests. [file 12909_2014_244_MOESM1_ESM.docx]

**Additional file 1: Questions used in the pre and post tests**

***Omar is 30-year-old new migrant who was born in Algeria. He comes to see you at the migrant health service because of general malaise over the past year. You decide to test him for hepatitis B.***

**1. You get the following results:**

- **HBsAg negative**
- **anti-HBs positive**
- **anti-HBc negative**

**Which is the correct interpretation of these results?**

1. He has been immunised in the past
2. He is a hepatitis B virus carrier
3. He has acute hepatitis B infection
4. He has resolved hepatitis B infection
5. He has chronic hepatitis B infection
6. I don't know

**2. If his test results had showed the following instead:**

- **HBsAg positive**
- **anti-HBs negative**
- **anti-HBc positive**

**Which would have been the best interpretation of these results?**

1. He has been immunised in the past
2. He is a hepatitis B virus carrier
3. He has acute hepatitis B infection
4. He has resolved hepatitis B infection
5. He has chronic hepatitis B infection
6. I don't know

**3. What test can you order to confirm if he has chronic hepatitis B infection?**

1. HBeAg
2. Anti-HBe
3. LFTs
4. HBV DNA viral load
5. Repeat HBsAg in 6 months
6. I don't know

**4. Which of the following is the most commonly used marker of current infection?**

1. HBsAg
2. HBeAg
3. Anti-HBs
4. Anti-HBc
5. Anti-HBe
6. HBV DNA
7. ALT
8. INR
9. I don't know

**5. Which of the following is the most useful to check if a hepatitis B vaccine has been effective?**

1. HBsAg
2. HBeAg
3. Anti-HBs
4. Anti-HBc
5. Anti-HBe
6. HBV DNA
7. ALT
8. INR
9. I don't know

**6. Which of the following is useful to differentiate immunity secondary to previous HBV infection and immunity secondary to vaccination?**

1. HBsAg
2. HBeAg
3. Anti-HBs
4. Anti-HBc
5. Anti-HBe
6. HBV DNA
7. ALT
8. INR
9. I don't know

|  |  |
| --- | --- |

**7. The appearance of which of the following signals a transition from high disease activity to immune control?**

1. HBsAg
2. HBeAg
3. Anti-HBs
4. Anti-HBc
5. Anti-HBe
6. HBV DNA
7. ALT
8. INR
9. I don't know

***Thomas is a new migrant from Papua New Guinea. He is 30 years old and was born in Port Moresby. He comes to see you because of nausea and weight loss. You decide to test him for hepatitis B.***

**8. You order hepatitis B serology and get the following results:**

- **HBsAg negative**
- **Anti-HBs positive**
- **Anti-HBc positive**

**Which is the correct interpretation of these results?**

1. He has been immunised in the past
2. He is a hepatitis B virus carrier
3. He has acute hepatitis B
4. He has resolved hepatitis B infection
5. He has chronic hepatitis B infection
6. I don't know

***Akbar is from Ethiopia and was told he had hepatitis B many years ago.***

***Tests showed the following:***

- ***HBsAg positive***
- ***Anti-HBc positive***
- ***HBeAg positive***
- ***Anti-HBe negative***
- ***LFT normal***

**9. Which stage of hepatitis B infection is Akbar at?**

1. Acute HBV infection
2. Resolved HBV infection
3. Chronic HBV infection, Phase 1
4. Chronic HBV infection, Phase 2
5. Chronic HBV infection, Phase 3
6. Chronic HBV infection, Phase 4
7. I don't know

***Six months later you check his serology again and find:***

- ***HBsAg positive***
- ***Anti-HBc positive***
- ***HBeAg positive***
- ***Anti-HBe positive***

10.Which stage of hepatitis B infection is Akbar at now?

1. Acute HBV infection
2. Resolved HBV infection
3. Chronic HBV infection, Phase 1
4. Chronic HBV infection, Phase 2
5. Chronic HBV infection, Phase 3
6. Chronic HBV infection, Phase 4
7. I don't know

|  |  |  |
| --- | --- | --- |
